# Sleep-Disordered Breathing in People with Multiple Sclerosis: Prevalence, Pathophysiological Mechanisms, and Disease Consequences

**DOI:** 10.3389/fneur.2017.00740

**Published:** 2018-01-15

**Authors:** Hanna A. Hensen, Arun V. Krishnan, Danny J. Eckert

**Affiliations:** ^1^Neuroscience Research Australia (NeuRA), Sydney, NSW, Australia; ^2^School of Medical Sciences, University of New South Wales, Sydney, NSW, Australia; ^3^Prince of Wales Clinical School, University of New South Wales, Sydney, NSW, Australia

**Keywords:** sleep disorders, sleep apnea, obstructive, central, pathophysiology, fatigue, multiple sclerosis, cognition

## Abstract

Sleep problems are common in people with multiple sclerosis (MS). Reported prevalence rates of sleep-disordered breathing (SDB) vary between 0 and 87%. Differences in recruitment procedures and study designs likely contribute to the wide variance in reported prevalence rates of SBD in MS. This can make attempts to compare SDB rates in people with MS to the general population challenging. Little is known about the pathophysiological mechanisms that contribute to SDB in people with MS or whether MS contributes to SDB disease progression. However, compared to the general obstructive sleep apnea (OSA) population, there are clear differences in the clinical phenotypes of SDB in the MS population. For instance they are typically not obese and rates of SDB are often comparable or higher to the general population, despite the high female predominance of MS. Thus, the risk factors and pathophysiological causes of SDB in people with MS are likely to be different compared to people with OSA who do not have MS. There may be important bidirectional relationships between SDB and MS. Demyelinating lesions of MS in the brain stem and spinal cord could influence breathing control and upper airway muscle activity to cause SDB. Intermittent hypoxia caused by apneas during the night can increase oxidative stress and may worsen neurodegeneration in people with MS. In addition, inflammation and changes in cytokine levels may play a key role in the relationship between SDB and MS and their shared consequences. Indeed, fatigue, neurocognitive dysfunction, and depression may worsen considerably if both disorders coexist. Recent studies indicate that treatment of SDB in people with MS with conventional first-line therapy, continuous positive airway pressure therapy, can reduce fatigue and cognitive impairment. However, if the causes of SDB differ in people with MS, so too may the optimal therapy. Thus, many questions remain concerning the relationship between these two disorders and the underlying mechanisms and shared consequences. Improved understanding of these factors has the potential to unlock new therapeutic targets.

## Introduction

Understanding the potential link between sleep disruption and multiple sclerosis (MS) due to a sleep disorder or other MS disease-related causes is important. Recent studies have demonstrated that “poor sleep” is more common in people with MS compared to the general population (1, 2). Poor sleep can worsen quality of life and may contribute to the frequently experienced consequences of MS including fatigue and impaired neurocognitive function. Disrupted sleep may also contribute to MS disease progression (3–6). Potential disease-related contributors to sleep disruption in people with MS include symptoms such as pain, spasms, bladder dysfunction, or anxiety. However, sleep disorders such as insomnia, restless legs syndrome, and sleep-disordered breathing (SDB) have also been documented in MS (7, 8). Nonetheless, it remains uncertain if sleep disorders are more common in people with MS compared to the general population (9). This review focuses on SDB in MS. A summary of what is known regarding its prevalence, potential underlying mechanisms and links between the consequences of MS and SBD and how these may further perpetuate disease severity are discussed.

The estimated prevalence of SDB in middle-aged adults in the community ranges between 10 and 50% in men and 3 and 20% in women (10–12). Untreated SDB is associated with multiple adverse consequences including poor sleep quality, excessive daytime sleepiness, cognitive impairment, increased risk of motor vehicle accidents, and adverse cardiovascular outcomes (13–15). Recent data on the prevalence of SDB in MS are conflicting (16–19). Thus, it remains uncertain if SDB is more common in MS than in the general population. However, it is clear that MS and SDB have shared consequences such as fatigue, neurocognitive impairment, and depression which may contribute to increased morbidity. As will be discussed, there are also bidirectional pathophysiological pathways that may contribute to disease progression for both disorders (6, 20, 21).

Sleep-disordered breathing and other sleep disorders are often underdiagnosed in MS (7, 22). This is possibly due to the fact that fatigue is generally accepted as being an “intrinsic” symptom of the disease rather than due to other potentially contributing causes such as sleep disruption (23). Thus, the treating clinician might be less likely to refer a patient with MS for polysomnography (PSG) compared to a person without MS. To what extent SDB contributes to the fatigue, neurocognitive impairment and disease progression in people with MS is not known. However, treating SDB may improve multiple aspects of MS. Recent studies that have included a relatively small number of participants have highlighted the potential beneficial role of treatment of SDB with continuous positive airway pressure (CPAP) to reduce fatigue in people with MS (24, 25).

In this review, current knowledge regarding the following key questions are addressed: In people with MS, how common is SDB? What are the risk factors and the pathophysiological causes? How will treatment influence key outcomes such as fatigue, neurocognitive function, balance, and disease severity/progression?

## How Common is SDB in MS?

Sleep studies in people with MS show conflicting results with regard to the prevalence of SDB. Indeed, the reported prevalence varies between 0 and 87% (6, 7, 16–19, 24–35). A summary of the studies is shown in Tables 1 and 2. Table 1 highlights studies with objective sleep data (i.e., PSG or equivalent) and Table 2 covers studies with subjective data (i.e., questionnaires).

**Table 1:** Sleep studies in people with multiple sclerosis.

% of SDB	Study	Year	*N* (♀)	Study type	Patient selection	AASM scoring criteria	EDSS [mean ± SD or (range)]	Age (years) (mean ± SD)	BMI (kg/m^2^) (mean ± SD)	SDB (%)	OSA/SDB^a^ (%)	CSA/SDB^a^ (%)	Disease-modifying therapy (%)
<20	Ferini-Strambi et al. (26)	1994	25 (12)	PSG-L	N	–	3.5 (1–5.5)	39.9	–	12	33	66	0
	Tachibana et al. (28)	1994	28 (16)	OXI and PSG-L	N	–	6.4 ± 2.0	42.3 ± 11	23 ± 3.8	7	50	50	–
	Kaynak et al. (27)	2006	37 (21)	PSG-U	N	1999	2.4 ± 1.4	37.4 ± 8.7	–	0	0	0	–
	Vetrugno et al. (29)	2007	6 (2)	PSG-L	Fatigued	–	2.4 ± 1	36.6 ± 11.2	26 ± 1.7	0	0	0	None
	Veauthier et al. (25)	2011	66 (45)	PSG-H	N	–	2 ± 1.8	43.2 ± 10	25♀	12	75	12.5	–
									26♂				
	Neau et al. (16)	2012	25 (15)	PSG-L	Fatigued	–	2.2 ± 1.7–4.1 ± 2.5^b^	39.7 ± 9.3–40.1 ± 11.2^b^	24.8 ± 4.6	0	0	0	48
	Chen et al. (18)	2014	21 (15)	RESP and PSG-L	N	2007	4.0 ± 2	29 ± 8.5	23.9 ± 1.0 (NF) 25.3 ± 3.1 (F)	0	0	0	–
	Lin et al. (34)	2016	19 (14)	RESP	EDSS (2–6)	2007	3.3 ± 1.2	56 ± 10	28 ± 8	16	0	100	84
>20	Kallweit et al. (30)	2013	69 (48)	RESP	Severely fatigued	2007	5.8 ± 1.4	49.8 ± 9.2	26 ± 4.9	41	94	6	–
	Kaminska et al. (17)	2012	62 (45)	PSG-L	N	1999	3.6 ± 1.8	47.3 ± 10.4	26 ± 5.1	58	100	0	69
	Braley et al. (35)	2012	48 (32)	PSG-L	PSG referred	2007	–	47.6 ± 10.8	32 ± 5.2	67	84	6	69
	Carnicka et al. (31)	2015	50 (35)	PSG-L	N	2007	2.5 (0–6.5)	40.3 ± 10.7	N/R	28	79	21	–
	Sater et al. (19)	2015	32 (24)	PSG-L	Fatigued/sleepy	2007	2.7 ± 1.8	45.7 ± 9.2	28.9 ± 6.4	38	92	8	None
	Braley et al. (6)	2016	38 (21)	PSG-L	Sleepy/CD	2007	3.4 ± 1.6	48.3 ± 10.1	N/R	87	100	0	50

*^a^Remaining percentages not mentioned here are made up by people with mixed sleep apnea*.

*^b^In patients with Epworth sleepiness scale score > 10, the mean EDSS was 4.1 ± 2.5 and age 39.7 ± 9.3; and in patients with Epworth sleepiness score < 10, EDSS score was 2.2 ± 1.7 and age 40.1 ± 11.2*.

*Sleep study type: PSG, polysomnography; PSG-L, in lab PSG; PSG-H, home-based PSG; PSG-U, unknown home or in lab PSG; OXI, oximetry; RESP, respirography. Patient selection: N, no specific selection except MS diagnosis; CD, cognitive dysfunction. Study design: all studies were prospective except Braley et al. (35) was retrospective. AASM, American Academy of Sleep Medicine; EDSS, expended disability status scale; BMI, body mass index; NF, non-fatigued; F, fatigued; SDB, sleep-disordered breathing; OSA, obstructive sleep apnea; CSA, central sleep apnea*.

**Table 2:** Screening questionnaires for OSA in people with multiple sclerosis.

Study	*N* (♀)	Patients selection	Disease duration (years) (mean ± SD)	Age (years) (mean ± SD)	BMI (kg/m^2^) (mean ± SD)	Questionnaire	High risk of OSA (%)	Official SDB diagnosis (%)
Dias et al. (32)	103 (74)	OPC	11.7 ± 8.9	45.8 ± 11.0	28 ± 6.5	STOPBANG	42 (28♀, 76 ♂)	2
Brass et al. (7)	2,375 (1,917)	Membership list MS society	16.3 ± 10.8	54.7 ± 12.4	>25 in 61%^a^	STOPBANG and Berlin	STOPBANG: 37	4
							Berlin: 37	
Braley et al. (22)	195 (128)	OPC	10.2 ± 8.2	47.1 ± 12.1	29.6 ± 7.4	STOPBANG	56	21
Ma et al. (33)	231 (135)	OPC	4.9 ± 2.2	40.2 ± 7.8	24.8 ± 4.6	STOPBANG	36	–

*^a^Mean BMI not provided, only percentage of people with BMI above 25 kg/m^2^ is available*.

*Patient selection: OPC, outside patient clinic; BMI, body mass index; SDB, sleep-disordered breathing; OSA, obstructive sleep apnea; high risk of OSA, >3 points on STOPBANG questionnaire or 2 positive categories on Berlin questionnaire*.

The variability in SDB prevalence between PSG studies in people with MS (0–87%) can be explained by differences in study design, study population and methodology. Most of the studies were not designed to measure the prevalence of SDB *per se*. Some studies are retrospective (35), or included only fatigued patients (16, 19, 30). The study populations vary greatly in characteristics such as age, disability level, body mass index (BMI), and female to male ratio. Regarding methodology the studies used different recording equipment and respiratory event scoring criteria. PSG technology has advanced significantly in recent years leading to increased sensitivity to detect respiratory events. The American Academy of Sleep Medicine scoring criteria have also been modified several times which have influenced respiratory event and SDB thresholds (36, 37).

The estimated prevalence of SDB based on questionnaire studies is more consistent and varies between 36 and 56% (7, 22, 32, 33). However, these data are somewhat difficult to interpret as they represent the proportion of people with a high risk of obstructive sleep apnea (OSA) rather than direct objective assessment. It is also unclear whether current OSA screening tools, such as the STOPBANG (38) and Berlin questionnaires (39), are appropriate in the MS population. Indeed, they were designed and validated to assess OSA risk in the general non-MS population and have not been validated in people with MS. Nonetheless, a positive STOPBANG screening (a score of 3 or more out of a possible 8) has a positive predictive value of between 75 and 85% to detect OSA in the general population (38, 40). If this is similar in the MS population, these studies (7, 22, 32, 33) indicate a high prevalence of OSA. However, these questionnaires may not be accurate in the MS population because tiredness plays a major role in both questionnaires. These questions may confound the ability to detect sleep-specific consequences in the context of MS where disease-related symptoms of fatigue and tiredness are common. Indeed, while fatigue and sleepiness are separate constructs these aspects may be difficult for some people with MS to separate in OSA screening questionnaires. Additionally, these questionnaires are based on well-known risk factors for OSA in the general population. However, it is uncertain whether these risk factors play the same key roles in the development of SDB in people with MS. Accordingly, studies that combine questionnaire with PSG data are needed to define the accuracy of these questionnaires in the MS population. Additional knowledge about MS specific risk factors for SDB is also required as outlined below.

In summary, data from studies conducted over the past 20 years indicate that SDB is common in MS but the varied study designs and populations tested make it challenging to make direct comparisons with population based studies in the general community.

## What are Possible Risk Factors for SDB in MS?

In order to determine possible risk factors for SBD in MS, we have separated PSG studies into two groups: (1) studies with a lower than 20% prevalence of SDB and (2) studies with a higher than 20% prevalence of SDB (Table 1). In the subsequent section, we compare these groups and individual studies by known risk factors for SDB including: age, BMI, sex, and the MS-specific expanded disability status scale (EDSS) score.

### Age

**Figure 1:**
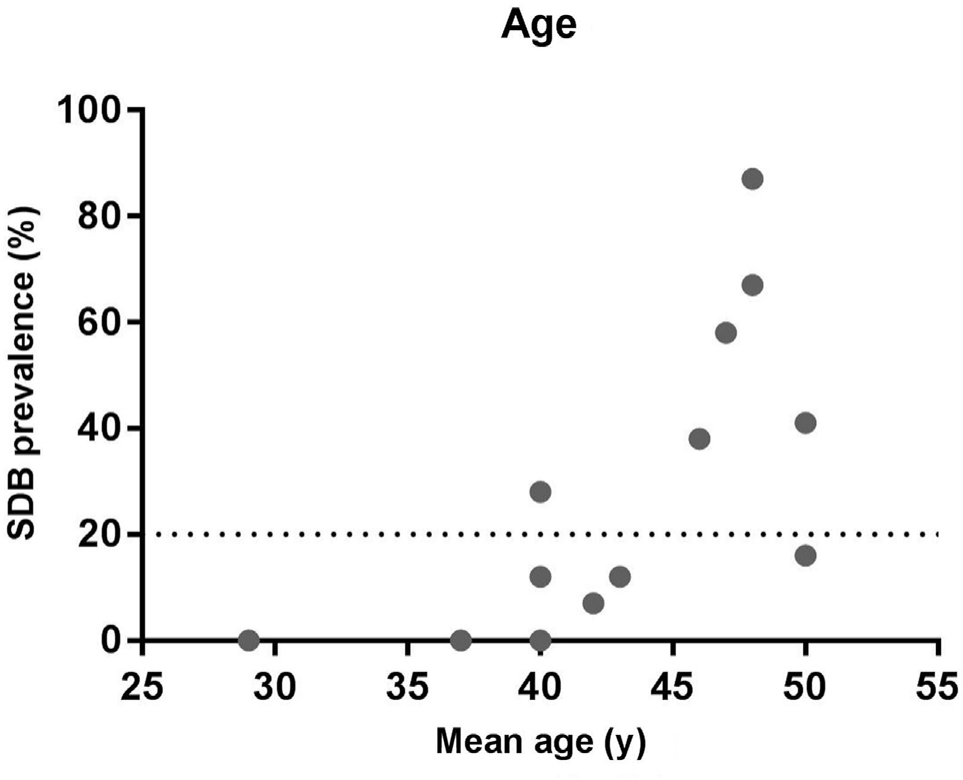
Relationship between sleep-disordered breathing (SDB) prevalence and age. The data show the prevalence of SDB and the mean age from the studies listed in Table 1.

The mean age of the participants in the studies that detected a high prevalence of SDB is typically 45 years or above with the exception of the Carnicka et al. (31) study in which the mean age was 40 years (Figure 1). Conversely, in the lower prevalence studies the mean age was below 43 years in all cases with the exception of our recent pilot study in which the mean age was 50 years (34). Thus, similar to the general population (11, 41), increasing age appears to be risk factor for SDB in the MS population.

However, based on the available knowledge, it is not possible to distinguish if the increasing age causes SDB *via* the same mechanisms in the general versus MS population or whether MS specific disease duration factors are involved.

### Body Habitus

**Figure 2:**
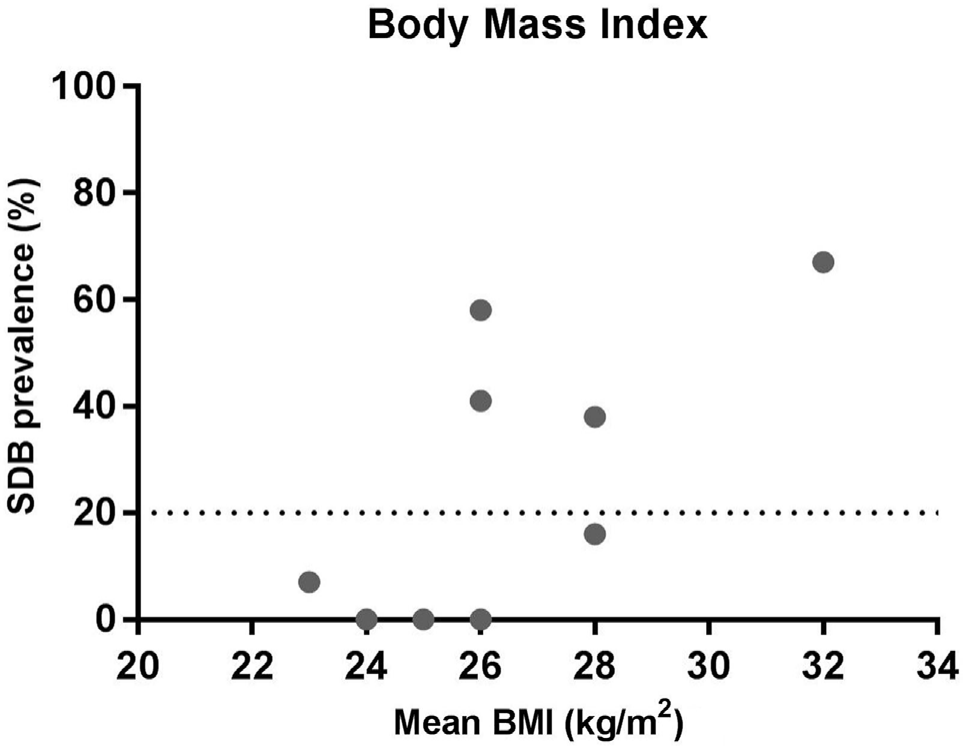
Relationship between sleep-disordered breathing (SDB) prevalence and mean body mass index (BMI). The data show the prevalence of SDB and mean BMI from the studies listed in Table 1 where BMI was reported.

While not all SDB studies in MS provide BMI data, in general, group mean BMI is less (BMI 23–28 kg/m^2^) in studies with a low prevalence compared to those with a higher prevalence of SDB (BMI 26–32 kg/m^2^) (Figure 2). Thus, similar to the general population (42), elevated BMI appears to be a risk factor in the MS population. However, with the exception of the Braley et al. study (35), the mean BMI for all of the MS studies is under 30 kg/m^2^. Indeed, in the studies of Kallweit et al. and Kaminska et al., the average BMI is 26 kg/m^2^, but the prevalence of SDB is 62 and 69%, respectively (17, 30). This is clearly very high for a non-obese population. Thus, while BMI still appears to be a risk factor for SDB in the MS population, many people with MS who have SDB have much lower BMI’s than the typical OSA patient population. Thus, the pathophysiological causes of OSA are likely to be quite different as outlined below.

### Sex Differences

In the general population, SDB is two to three times more common in men compared to women (43). MS on the other hand is more common in women (3:1). Thus, almost all of the populations in which polysomnographs have been performed involve more women than men. Thus, it is important to consider the female to male ratios when interpreting the prevalence of SDB in MS. Unfortunately, most studies do not specify the prevalence of SDB by gender. However, in the two studies that do (25, 30), the prevalence of SDB is higher in men compared to women. In the Kallweit et al. study, the prevalence was 33% in women and 60% in men (41% overall); and in the Veauthier et al. study, 11% of women and 14.2% of men (12% overall) had SDB. Thus, while the number of studies is small, similar to the general population, male gender is a risk factor for SDB in the MS population. Nevertheless, it is noteworthy that some studies show such high rates of SDB even though the majority of the study populations are pre-menopausal women.

### MS Disability

**Figure 3:**
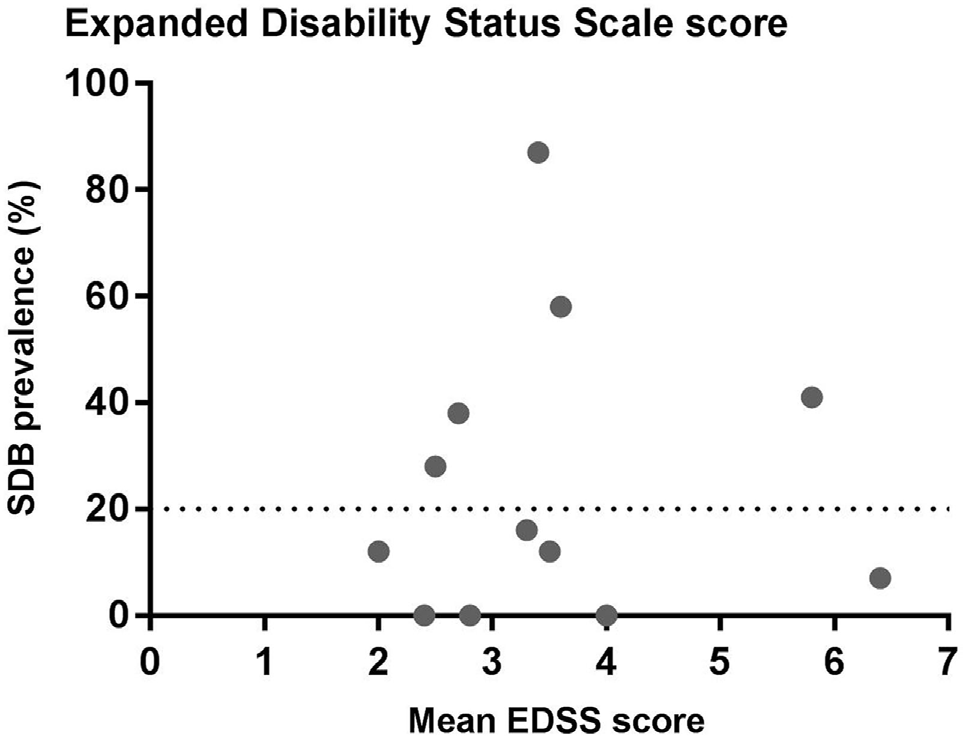
Relationship between sleep-disordered breathing (SDB) prevalence and expanded disability status scale score (EDSS). The data show the prevalence of SDB and the mean EDSS scores from the studies listed in Table 1.

Multiple sclerosis disability is most commonly measured using the well-established EDSS score. EDSS varies from 0 (normal neurological examination) to 10 (death due to MS) (44) (Figure 3). There is substantial variation in mean EDSS scores in the studies that have investigated SBD in MS. There is no clear relationship between the prevalence of SDB and mean EDSS score based on the existing studies (Figure 3). However, this does not necessarily mean that MS severity does not influence SDB. The EDSS score is a good measure of clinical and functional disability, but it is heavily weighted toward motor and ambulatory deficits. Its correlation with lesion load and brain atrophy on magnetic resonance imaging (MRI) varies (45, 46). Thus, the extent to which other disease severity measures (e.g., lesion load and brain atrophy on MRI) influences the prevalence of SDB in people with MS is a valuable objective to assess in future studies.

In summary, standard SDB risk factors (e.g., age and BMI) also appear to play a contributing role in people with MS. However, in general, people with MS and SDB tend not to be obese. The extent to which MS specific factors like disability and severity influence the prevalence of SDB is unclear.

## Pathophysiological Mechanisms of SDB in MS and Potential Bidirectional Links

In this section, we attempt to address the following key questions: What are the causal pathways of SDB in people with MS? Are they the same as in the general population? How do SDB and MS influence each other?

To further explore these questions, a brief overview of the current understanding of the pathophysiology of SDB (OSA and central sleep apnea [CSA]) in the general population is highlighted below.

### Obstructive Sleep Apnea

In OSA, there is an absence of airflow due to collapse of the upper airway, despite ongoing respiratory effort. Apneas and hypopneas are often associated with cortical arousals. The pathophysiological causes of OSA vary markedly between individuals. There are anatomical and non-anatomical contributors. The key pathophysiologic causes are: (1) an anatomically narrow or collapsible upper airway, (2) insufficient upper airway dilator muscle responsiveness during sleep, (3) a low respiratory arousal threshold, and (4) having an oversensitive ventilatory response system (high loop gain) (47–49). Inflammation state can also influence OSA (50, 51).

Major risk factors for OSA are aging, male sex, and obesity. The underlying mechanisms remain largely unclear, although other mediating mechanisms likely contribute *via* the pathophysiological causes highlighted above.

### Central Sleep Apnea

In CSA, there is an absence of airflow, without respiratory effort. Thus, in CSA there is a fundamental issue with respiratory control during sleep. Briefly, the primary centers for central respiratory control are based in the pons and the medulla. These centers receive input from a variety of sources within the body including: the cortex (voluntary control), the limbic system (emotional stimuli), stretch receptors in the lungs, receptors for touch temperature and pain, receptors in muscles and joints, peripheral O_2_, CO_2_, and H^+^ chemoreceptors located at the bifurcation of the carotid arteries, and central CO_2_ and H^+^ chemoreceptors located at ventral surface of the medulla. Most of these inputs are capable to influence breathing during wakefulness, but are absent or decreased during sleep. Chemical control *via* the chemoreceptors is also decreased in sleep. However, it is the key regulator of breathing during sleep (52). There are several manifestations of CSA and the precise pathophysiological mechanisms vary, but in all instances unstable ventilatory drive is the principal underlying mechanism. There are three levels in the control of breathing during sleep where impairment could lead to CSA. There is (1) primary impaired central drive, for example caused by a lesion in the brain stem affecting the respiratory control center, (2) impaired output of central drive due to abnormalities located anywhere from upper motor neurons down to the respiratory muscles, for example caused by neuromuscular dysfunction, or (3) the central drive feedback loop is impaired, for example caused by prolonged blood circulation time in people with heart failure, resulting in a mismatch between arterial blood gas concentrations and respiratory controllers (53). Thus, impairment in one or more of these levels of the control of breathing can lead to CSA.

### Bidirectional Relationships

**Figure 4:**
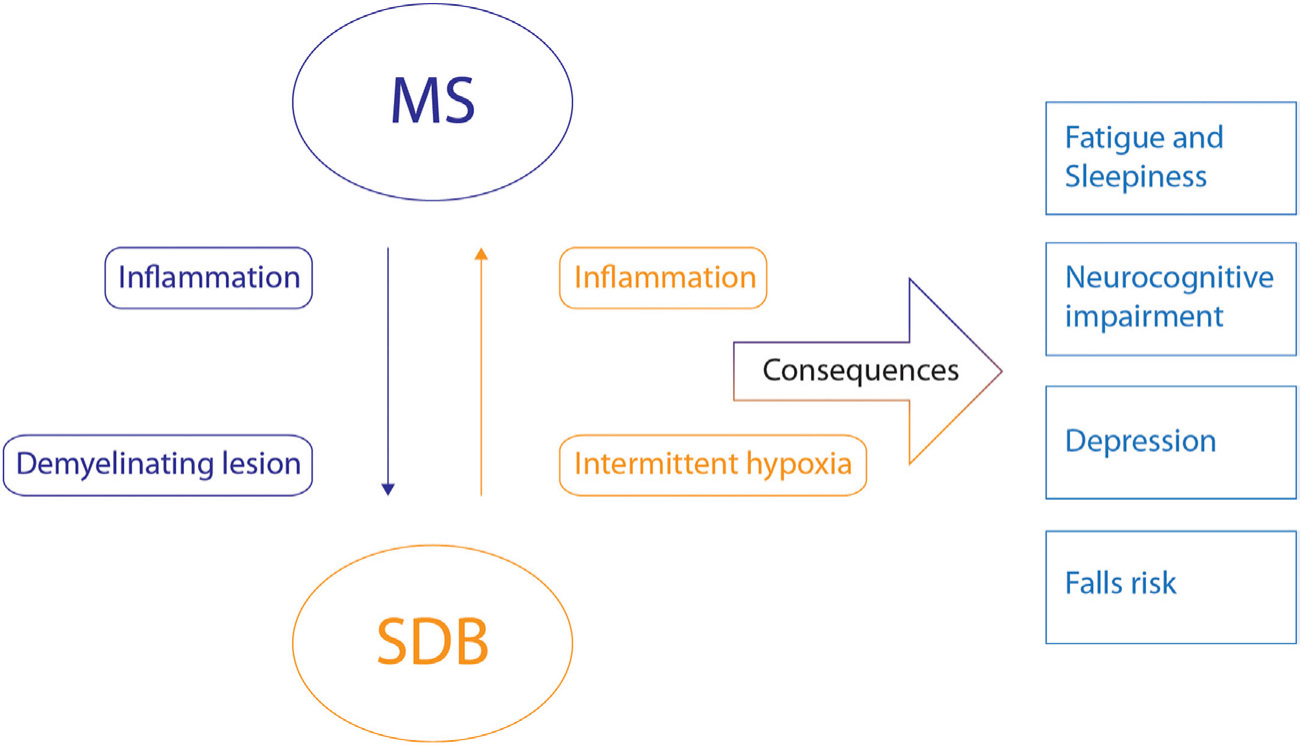
Potential bidirectional relationships between multiple sclerosis (MS) and sleep-disordered breathing (SDB) and their shared consequences. The potential bidirectional pathophysiological relationship between MS and SDB is indicated by the arrows with the corresponding mechanisms. Inflammatory state and demyelinating lesions of MS may induce or influence SDB. Intermitted hypoxia and inflammation caused by SDB may also influence MS. The consequences that these disorders share are indicated on the right. The combination of these disorders will likely negatively influence these consequences.

To date, no studies have been conducted to characterize the phenotypic causes of SDB in people with MS. There are several possibilities and potential bidirectional relations that may create a vicious cycle between both disorders as highlighted in Figure 4.

#### MS As a Contributor to SDB

Demyelinating lesions of MS in the brain stem and spinal cord could influence or evoke CSA *via* impairment of ventilatory drive. Lesions in the brain stem may influence primary central ventilatory drive centers directly or key components of the central breathing control feedback loop. Lesions in the spinal cord could also cause impairment of motor neurons that control the respiratory muscles to reduce ventilatory drive output. In accordance with this theory, Braley et al. have shown that the mean apnea hypopnea index and central apnea index of people with brain stem lesions in MS was significantly higher than controls or people with MS without known brain stem lesions (35). Furthermore, in the MS population studied by Lin et al., the only SDB that was detected was CSA and not OSA which is very rare in the non-MS population in the absence of key comorbidities such as heart failure. Other neurological disorders that affect brain stem function such as tumors (54, 55), hemorrhages, ischemia (56, 57), and Arnold-Chiari malformation (58), can also influence or cause SDB. However, in the studies listed in Table 1, the majority of people had OSA not CSA. This could be explained, because demyelinating lesions in the brain stem and spinal cord could also result in impaired upper airway muscle responsiveness and an unstable ventilatory response system, both of which contribute to OSA. Thus, demyelinating lesions in the brain stem and spinal cord are potentially important contributors to SDB in MS.

Second, inflammation in MS may also play a role in the occurrence of SDB. MS is an autoimmune disorder associated with changes in cytokine levels (59). How proinflammatory cytokines influence SDB is not known. However, systemic cytokine levels of TNF-a, IL-6, C-reactive protein, IL-1b, reactive oxygen species, and adhesion molecules are increased in people with OSA (50, 60). In MS, levels of TNF-α, IL-6, and IL-1b amongst others are also elevated. Anti-inflammatory therapy with entracept (a TNF α antagonist) can improve OSA severity in the general population (51). This suggests that disease-modifying therapy with anti-inflammatory agents used in MS may also have a beneficial effect on OSA. However, to our knowledge there have been no studies conducted to investigate the potential relationship between inflammation in SDB and MS.

#### SDB As a Contributor to MS

As highlighted, chronic inflammation is a feature of both SDB and MS. Thus, the proinflammatory state caused by SDB may interact with the inflammatory process taking place in MS and contribute to disease progression or relapse frequency. However, this theory has not been formally investigated.

The second factor that can play an important role in the progression of MS due to SDB is intermittent hypoxia. One of the potential factors attributed to lesion progression in MS is “virtual hypoxia,” which is a state of reduced oxygen consumption and energy failure in conditions of normal blood and oxygen supply leading to cell death. “Virtual hypoxia” is caused by damaged mitochondria, likely attributed to chronic oxidative injury. Inflammation of the brain tissue drives microglia and macrophage activation, resulting in oxidative stress, causing mitochondrial injury, making it a vicious cycle of tissue destruction and energy failure (61, 62). When brain tissue is affected by “virtual hypoxia,” additional intermittent hypoxia due to SDB may amplify neurodegeneration. Indeed, studies have shown that there are increased MS lesion loads in the “watershed” areas of the brain and spinal cord, which are areas that are on the boundaries between the blood supply of the cerebral arteries and therefore less well perfused and most vulnerable to reduced blood flow or reduced oxygen. This suggests that hypoxia may play an important role in lesion pathology (63). In addition, animal studies indicate that demyelination can be reduced or eliminated by increasing inspired oxygen to alleviate transient hypoxia (64). This progression of disease due to mitochondrial injury and energy failure is thought to play an especially important role in the progressive phase of the disease. Progressive MS is divided in primary progressive (being progressive from the moment of diagnosis) and secondary progressive (being progressive after the initial relapsing remitting phase). Progressive MS commonly affects the middle aged population (65). This is important since middle aged adults are at high risk of SDB. There are only few treatments available for the progressive phase of the disease and anti-inflammatory drugs yield little improvement (61). Thus, diagnosing SDB and effectively treating SDB to restore oxygenation in the brain may play a pivotal role in slowing progression in this phase of the disease. This is a priority for future research.

## Shared Consequences of SDB and MS

Sleep-disordered breathing and MS have shared consequences, as shown in Figure 4. These consequences may be synergistic if both disorders are present. Important shared consequences include: fatigue/sleepiness, neurocognitive dysfunction, depression, and falls risk. Although not covered in this review, there may also be other shared links between cardiovascular risk, inflammation, SDB, and MS (66–68). Diagnosis and treatment of SDB in afflicted people with MS may help alleviate these common consequences as discussed below.

### Fatigue and Sleepiness

Fatigue is a common symptom in MS. Indeed, fatigue is often the most debilitating symptom of MS and has a major impact on quality of life (20, 21, 23, 69). There have been multiple studies on fatigue in MS including various strategies on quantification, its potential origin and treatment. However, the pathophysiology still remains to a large extend unknown. Kos et al. (70) divided fatigue in MS into primary fatigue and secondary fatigue. Primary fatigue may be due to centrally mediated processes, like demyelination in the central nervous system and immune activity. Factors that may influence secondary fatigue are sleeping problems, pain, depression, reduced activity, psychological functioning, and medication use.

It is important to note that fatigue and sleepiness are two distinct but potentially interrelated symptoms that often coexist. Chervin et al. have shown that patients with moderate to severe OSA report problems with fatigue, tiredness, and lack of energy more frequently than sleepiness (57, 61, 62, and 47%) (71). Hossain et al. assessed 283 patients with sleep disorders and in this cohort 64% reported pathological fatigue without overlap of sleepiness and only 4% reported sleepiness without overlap fatigue. This suggests that fatigue and sleepiness can be independent manifestations of sleep disorders (72). The extent to which SDB plays a role in fatigue in MS has been assessed in several studies. Veauthier et al. studied 66 MS patients who underwent PSG and completed the modified fatigue impact scale (MFIS) to quantify fatigue severity. There were eight patients with SDB, of which seven were fatigued, defined as a score > 45 on the MFIS. The people with SDB also had higher scores on the MFIS compared to fatigued people without SDB (25). Kaminska et al. found a relationship between severe OSA (apnea hypopnea index > 30 events/h sleep) and severe fatigue in people with MS. The mean fatigue severity scale (FSS) scores were also significantly higher in people with MS and severe OSA compared to those without severe OSA (17).

In addition, some studies have assessed the risk of OSA, using the STOPBANG questionnaire, and its relationship with fatigue. Dias et al. found a significant correlation in STOPBANG scores and FSS scores in males but not in females (32). Braley et al. demonstrated that OSA is a significant predictor of fatigue when adjusted for other clinical- and sleep-related factors for fatigue (22). Brass et al. demonstrated an odds ratio of a STOP BANG > 3 to having a FSS > 36 is 1.85, consistent with an association between OSA and fatigue (7). Thus, it appears that SDB has a negative effect on fatigue in people with MS.

Fatigue is a very difficult symptom of MS to treat. All current therapies are either ineffective or only partially effective (73). To date, there have only been a few studies to examine whether treatment of SDB improves fatigue in MS. Kallweit et al. treated 6 MS patients with OSA with CPAP therapy for 6 months with a daily average adherence of >5h per night. Measures of sleepiness with the Epworth sleepiness scale (ESS) and fatigue with the FSS were assed at baseline and after 6 months. The apnea hypopnea index decreased from 39 to 5 events/h sleep. ESS scores did not show any significant change (9.8–9.5), but there was a significant decrease in FSS scores from 5.8 to 4.8. However, the FSS score remained pathologic (>4) in all patients (30). Cote et al. also treated MS patients with OSA with CPAP and compared ESS and FSS scores before and after 3 months of therapy. ESS scores decreased significantly with treatment from 8.9 to 5.4 and FSS scores decreased from 5.0 to 4.3. However, this change was not statistically significant (24). Larger studies are needed in people with MS and SDB to quantify the effects of treatment on fatigue in people with MS.

### Neurocognitive Dysfunction

In the general population, SDB can result in neuropsychological impairment (15, 74). Changes in brain structure have also been demonstrated in people with OSA and impaired cognitive function using imaging techniques (75, 76). Neuropsychological domains influenced by OSA include: attention/vigilance, executive function, and memory. The ethology of cognitive dysfunction in OSA is believed to be multifactorial with sleep fragmentation and hypoxemia as key contributors. These factors may have similar or greater effects in people with MS. Nocturnal hypoxia in OSA can cause neuroimaging and neuropathological abnormalities in regions of the cortex and axons that are similar to cognitive domains affected in MS (77). There has been only one study conducted to investigate the relationship between cognitive function and OSA in people with MS. Braley et al. assessed 38 MS patients from an outside patient clinic that asked about sleep or cognition during routine visits. Participants were evaluated by a neurologist, neuropsychologist and an overnight PSG was conducted. Cognitive function testing was performed using the validated 90-min battery, minimal assessment of cognitive function in MS. Regression models demonstrated an association between several components of neuropsychological function (attention and working memory) and oxygen desaturation index, minimum oxygen saturation, and respiratory disturbance index. An association between verbal memory and response inhibition and sleep quality parameters was also shown (6). Similar findings have been demonstrated in the general population (78, 79).

Cognitive function in people with MS and OSA could potentially be improved with CPAP therapy. Beneficial effects of CPAP on cognitive performance have been shown in the non-MS OSA population in some (75, 76) but not all studies (74). Given that oxygen levels may play a key role in the decrease in cognitive function, oxygen therapy may also be beneficial, either indirectly *via* reductions in SDB or directly *via* restoration of oxygenation (80). Braley et al. have a randomized controlled trial on the effects of CPAP on cognitive function in MS patients with OSA that is currently underway (NCT02544373). The results will be of great importance to evaluate the potential need for early diagnosis of SDB and the influence of treatment on cognitive function. There are currently few options to improve cognitive function in MS. However, immunomodulatory therapy may slow and reverse MS-related cognitive dysfunction and have a positive effect on OSA severity in MS (81, 82).

### Depression

Depression is common in people with MS, with an estimated annual prevalence of 20% and lifetime prevalence as high as 50% (83). Depression is more common in people with SDB compared to the general population. The estimated prevalence of depression in people from the general community who have OSA is between 17 and 22% and in clinical population studies ranges between 21 and 41% (84). For both disorders it is to a large extent unknown what their role is in the causation of depression. However, given the bidirectional relationship between sleep and the brain, it is likely that people with MS who experience sleep disruption due to SDB will be at higher risk of depression.

### Falls Risk

Falls frequently occur in people with MS. Several studies have shown that 50–60% of people with MS report falling at least once in a 2- to 6-month period (85–87). Medical care is often required. Important factors that increase the risk of falling in people with MS are loss of balance, impaired gait, use of a walking aid, leg weakness, visual impairment, reduced cognitive function, and fatigue (88).

There is growing evidence that poor sleep and sleep disorders can increase falls risk in the elderly independent of other factors (89–91). Since factors that increase the risk of falling in elderly are to a large extend similar to the ones in MS (like reduced balance, visual impairment, cognitive decline, and the use of a walking aid), poor sleep might have an equivalent impact on people with MS. In young adults postural sway, a measure of balance, significantly increases after sleep deprivation (92, 93). Excessive daytime sleepiness defined as a score > 10 on the ESS is also an independent risk factor for falls (94). Given that sleepiness is common in people with moderate to severe SDB, sleepiness due to SDB in people with MS may increase falls risk. There have only been a few studies that have examined the relationship between SDB and balance control. A recent study by Degache et al. noted an association between overnight hypoxia and postural instability (95). Two additional studies also showed an altered gait pattern in people with OSA compared to healthy controls (96, 97). The precise mechanism as to how falls risk increases due to poor sleep is unknown, but the combination of MS and SDB may have an additive effect in increasing the risk of falls. Therefore, given that SDB is a modifiable factor, treatment of SDB may reduce the risk of falling in people with MS.

## Summary and Future Research Directions

It remains uncertain how common SDB is in people with MS. However, many of the existing studies indicate that it is quite common despite the female predominance and typical lack of obesity in the MS population. Understanding the mechanisms of SBD in people with MS for which there are multiple plausible possibilities, may provide novel targets for therapy. Given the shared links between the two disorders and supported by several initial studies, improvements in sleep by treating SDB has the potential to reduce the burden of several of the key shared consequences and may also reduce MS disease progression. These are all important topics for future research.

## Author Contributions

HH drafted the manuscript, and all authors provided important intellectual input and contributed to the final version.

## Conflict of Interest Statement

HH and AK do not have any conflicts to declare. DE has a Cooperative Research Centre Project Grant which is cosupported by the Australian Government and an industry partner (Oventus Medical) and serves as a consultant for Bayer.

## Abbreviations

BMI, body mass index; EDSS, expanded disability status scale; ESS, Epworth sleepiness scale; FSS, fatigue severity scale; CPAP, continuous positive airway pressure; CSA, central sleep apnea; MFIS, modified fatigue impact scale; MRI, magnetic resonance imaging; MS, multiple sclerosis; OSA, obstructive sleep apnea; PSG, polysomnography; SDB, sleep-disordered breathing.
